# Breaking Barriers: Exploring Patient Satisfaction With the U.S. Healthcare System Among Iranian and Afghan Immigrants With Limited English Proficiency

**DOI:** 10.1111/1475-6773.70027

**Published:** 2025-08-12

**Authors:** Sara Imanpour, Rifat Sultana, Victoria Williams

**Affiliations:** ^1^ School of Public Affairs Pennsylvania State University Harrisburg Pennsylvania USA; ^2^ School of Business Rhode Island College Providence Rhode Island USA

**Keywords:** immigrants, limited English proficiency (LEP), satisfaction of care

## Abstract

**Objective:**

To explore the satisfaction of limited English proficiency (LEP) Farsi‐ and Dari‐speaking patients with the U.S. healthcare system using a qualitative approach.

**Study Setting and Design:**

We employed a grounded theory approach to analyze qualitative data collected from five focus groups involving 25 Farsi‐ and Dari‐speaking immigrants with LEP.

**Data Source and Analytical Sample:**

A total of 25 individuals with LEP participated in the focus group sessions, which were transcribed and analyzed using grounded theory methodology.

**Principal Findings:**

Two primary categories influencing satisfaction with care emerged: systemic factors and individual factors. Individual factors encompassed cultural beliefs, cross‐contextual comparisons, experiences of misdiagnosis, and language barriers. Systemic factors, including discrimination, the high cost of care, the complexity of the U.S. healthcare system, and a pharmaco‐centric approach to care, were found to negatively impact satisfaction among immigrants with LEP.

**Conclusions:**

Although many Farsi‐ and Dari‐speaking individuals with LEP expressed satisfaction with the structured aspects of the U.S. healthcare system, dissatisfaction with healthcare providers and interpersonal interactions persisted. Addressing these issues will require targeted interventions to enhance trust, communication, and cultural competency in healthcare delivery.

**Summary**

- What is known about this topic
  ○ Immigrants in the U.S. generally report lower satisfaction with healthcare services due to a lack of cultural and linguistic competency within the healthcare system.
  ○ Within the immigrant population, the experiences of individuals with limited English proficiency (LEP) remain underexamined, despite their significant language barriers in accessing and receiving care.
- What this study adds
  ○ U.S. immigrant research often centers on Latino populations and Spanish‐language access, but it is vital to include culturally and linguistically diverse groups, especially speakers of less common languages.
  ○ Although immigrants with LEP express overall satisfaction with the healthcare system, their satisfaction declines during clinical encounters due to cultural mismatches, language barriers, misdiagnoses, and cross‐contextual comparisons.

## 1 Introduction

Patient‐centered care is conceptualized in various ways throughout the literature; however, several foundational principles remain consistently recognized [1]. At its core, patient‐centered care emphasizes understanding and addressing the patient's experience within their broader psychosocial context. This approach calls for the active engagement of patients in care decisions that align with their values and preferences, while fostering a shared understanding of clinical concerns [2]. Effective clinical decision‐making within patient‐centered care must also be informed by the best available evidence and tailored to the patient's needs and the realities of the healthcare setting, as outlined by the U.S. National Cancer Institute [2, 3].

Improving patient‐centered care requires coordinated efforts across multiple, interconnected levels—including patients, clinicians, healthcare organizations, and policy systems [2, 4, 5]. Barriers at any of these levels can impede effective communication and compromise the quality of care. To address these challenges, comprehensive strategies such as workflow redesign and strong institutional support are essential [5, 6]. Importantly, Epstein et al. note that while interventions aimed at enhancing communication can improve patient‐centered dialogue, their influence on broader clinical and psychosocial outcomes may be limited [7].

Effective implementation of patient‐centered care not only hinges on communication but also closely intersects with patient satisfaction—an essential, although often imperfect, indicator of healthcare quality [5, 6, 7]. Patient satisfaction is a widely used measure of healthcare quality, but its relationship with clinical outcomes and care quality is complex. While overall satisfaction is high among most patients [8, 9], it may not accurately reflect the quality of care received, especially for immigrants [10]. The patient–provider relationship is the strongest predictor of overall satisfaction [8, 9]. Communication plays a crucial role, and providing clear information and properly identifying healthcare personnel positively impacts patient satisfaction [11, 12]. Trant et al. found that patients report higher satisfaction with care when their communication style aligns with that of their healthcare provider [13]. Studies have consistently shown that immigrant patients report lower levels of satisfaction with healthcare services than their native‐born counterparts [14, 15, 16]. Lanza et al. highlighted that suboptimal patient–physician interactions are a key predictor of dissatisfaction, particularly among Latina immigrants [17]. Several factors contribute to this disparity, including extended waiting times, language barriers, and cultural differences that hinder effective communication and understanding [18, 19]. Addressing these barriers is critical to enhancing both patient experiences and health outcomes among “hard‐to‐reach” immigrant populations, such as Farsi‐ and Dari‐speaking immigrants, who remain underrepresented in the existing literature [20].

Healthcare encounters are pivotal interactions between patients and healthcare providers that form the basis of healthcare delivery [21, 22]. However, negative encounter experiences can occur that impact patient satisfaction and well‐being [23]. Research on the healthcare experiences of immigrants reveals significant challenges in accessing and utilizing health services. Immigrants face barriers such as language difficulties, lack of insurance, and limited knowledge about available services [24, 25, 26, 27], which may have an impact on their overall satisfaction with care. For example, Dastjerdi found that language barriers and limited knowledge of the Canadian healthcare system negatively impacted Iranian immigrants' ability to access care in Canada, highlighting similar challenges faced by Middle Eastern immigrant populations more broadly [28]. Understanding and improving healthcare encounters is crucial for patient‐centered care, improving patient satisfaction with care, and positive health outcomes [29].

Over 25 million individuals in the United States (U.S.) are classified as having limited English proficiency (LEP); this population is projected to increase in number in the coming years [30]. Empirical evidence has consistently demonstrated that LEP individuals encounter significant barriers to accessing healthcare services, experience suboptimal health outcomes, possess inadequate insurance coverage, and report a diminished quality of life [26, 30, 31]. Despite these challenges, the voices and experiences of LEP populations remain critically underrepresented in public health research, resulting in a persistent gap in understanding and addressing their unique healthcare needs.

The U.S. has experienced a significant increase in immigration from Middle Eastern countries, particularly Afghanistan and Iran, which speak Farsi and Dari. This trend has been especially notable following the Taliban's takeover of Afghanistan in 2021 and the intensification of economic hardships in Iran due to prolonged U.S. sanctions. According to Montalvo and Batalova, the Afghan immigrant population in the U.S. increased from approximately 54,000 in 2010 to 195,000 in 2022 [32]. Similarly, data from Lai and Batalova show that in 2019, 385,000 Iranian immigrants were residing in the United States, with 36% reporting LEP [33]. Given that many of these immigrants arrived later in life, they often face significant challenges navigating the U.S. healthcare system due to language barriers. Shared cultural and linguistic backgrounds further compound these challenges, particularly among Iranian and Afghan immigrants.

Studying “hard‐to‐reach” populations is essential due to their heightened vulnerability in host countries and the unique challenges they face—challenges often unshared by other subgroups [20]. In the United States, efforts to improve health outcomes frequently overlook the specific barriers encountered by these marginalized groups, despite their significance within the broader American population. Achieving equitable, high‐quality care requires that the needs of these understudied populations be systematically addressed [34]. “Hard‐to‐reach” populations are typically defined by their limited participation in research or public health programs, often stemming from geographic isolation, socioeconomic disadvantage, or linguistic and cultural barriers [20, 34]. Afghan and Iranian immigrants, for instance, constitute a growing demographic in the United States yet remain underrepresented in health research. Their use of Farsi and Dari—languages rarely spoken in the United States—further complicates their access to care and inclusion in research, reinforcing the critical need to elevate their perspectives in healthcare policy and practice [35, 36].

Previous studies have identified a link between language barriers, immigration status, and dissatisfaction with care [13, 37, 38, 39]. While it is well‐established that language barriers contribute to dissatisfaction with the healthcare system [39], the underlying causes of this dissatisfaction—particularly among Middle Eastern immigrants with LEP—remain unclear. By examining these experiences in greater depth, policymakers may be able to develop more targeted and nuanced approaches to address this issue, ultimately improving healthcare access for minority populations. Such efforts could also advance the objectives of Healthy People 2030 in reducing health disparities within immigrant communities [40].

Therefore, this research seeks to address the following question: What factors impact satisfaction with the care received during a healthcare encounter among Afghan and Iranian immigrants with LEP? We selected a sample of “hard‐to‐reach” ethnic minorities—a population that is expected to grow in the United States. Studying their experiences offers valuable insights into the unique healthcare challenges faced by Afghan and Iranian immigrants with LEP, a subgroup that remains critically understudied.

## 2 Methodology

We adopted an epistemological and exploratory qualitative research approach to capture participants' stories and experiences [41]. Grounded theory was used to generate new insights into participants' interactions with the U.S. healthcare system [41, 42]. Furthermore, Lincoln and Guba argue that the only way to perceive social reality is from the perspective of the individuals who are embedded within it [43]. Therefore, we embraced this position (social constructivism) to guide our role as active participants throughout the data collection and analysis processes.

We utilized the grounded theory method to analyze the dataset [42], which is the preferred approach when little to no information is available about a particular phenomenon [44]. Given the limited understanding of dissatisfaction with care among immigrant populations with LEP, grounded theory provided a framework to generate new insights into their experiences within the U.S. healthcare system. This approach was particularly valuable for exploring the perspectives of ethnic minorities with language disabilities.

## 3 Participants

We recruited LEP participants by drawing from a pool of individuals involved in prior research [26, 45] and by posting recruitment flyers on social media pages, including the Facebook groups *Iranians of America* and *Afghan‐American Community*. The inclusion criteria were: (1) having LEP, (2) immigrating from Iran or Afghanistan to the United States, and (3) residing in the United States for less than 10 years. Individuals residing in the United States for over a decade may be more culturally and linguistically acculturated, which could make their experiences less representative of the target population for this study. Participants under the age of 18 and those who had lived in the United States for more than 10 years were excluded. In total, 25 Farsi‐ and Dari‐speaking participants were selected, and five focus groups (Table 1) were conducted after receiving IRB approval (00025988). Each participant was placed in a focus group based on their availability. Participants were recruited from across the United States, rather than from a single geographic region. We used a combination of convenience and homogeneous sampling. Convenience sampling was employed due to accessibility and voluntary participation, while homogeneous sampling ensured that all participants shared key characteristics relevant to the study, including language spoken (Farsi or Dari), immigrant background, and LEP.

**TABLE 1:** Focus group lay‐out.

Focus group numbers	Number of participants	Characteristics of the focus group	Duration of the focus group	Setting
Focus group 1	5	3 Iranian and 2 Afghan	80 min	All participants via FaceTime
Focus group 2	5	5 Iranian	70 min	4 Participants via FaceTime and 1 audio phone call
Focus group 3	5	4 Iranian and 1 Afghan	50 min	All participants via FaceTime
Focus group 4	6	5 Iranians and 1 Afghan	90 min	5 participants via FaceTime and 1 participant via audio phone call
Focus group 5	4	2 Iranian and 2 Afghan	60 min	2 participants via FaceTime and 2 via phone calls

## 4 Data Collection

Focus groups were chosen as the method of data collection because they rely on interactions between participants to generate data [46]. The first author moderated each focus group session, fostering an environment where participants connected well and appeared to enjoy discussing the topic with one another. Each session began with a welcome statement and overview of the topic and guidelines to ensure a productive discussion. These guidelines emphasized that there were no right or wrong answers, that audio and video recordings were solely for transcription purposes, and that participants should respect each other's opinions, even in cases of disagreement.

During the sessions, 15 questions were posed, focusing on participants' experiences of care in the host country's healthcare system, communication barriers, navigating the system, potential ways to improve satisfaction with received care, barriers to accessing care, and overall trust in the U.S. healthcare system. As recommended by Krueger, the focus group questions were developed based on our overarching research questions to ensure alignment between the study's objectives and the data collection process [46]. We employed a semi‐structured interview format, which provided the flexibility for participants to elaborate on their experiences while allowing the moderator to explore emergent topics through probing questions. This approach facilitated in‐depth, open‐ended dialogue and generated rich qualitative data [46]. After conducting five focus groups, the team determined that the data had reached saturation. At the beginning of each focus group session, the first author invited participants to choose their own pseudonym. In cases where participants were unable to come up with a name, the first author provided a list of suggested pseudonyms for them to select from.

All sessions were conducted in Farsi or Dari—languages spoken fluently by the first author—which facilitated communication and helped build rapport with participants. Each focus group lasted approximately 70–90 min, allowing ample time for in‐depth discussion. All sessions were conducted remotely via FaceTime or phone call, ensuring accessibility for participants. All focus group sessions were audio recorded using a secure voice recording application and stored on the first author's password‐protected computer. During each focus group, the first author took detailed field notes to document participants' emotions, body language, and overall engagement during the discussion. For participants who joined via phone and could not be visually observed, the first author made detailed notations of paralinguistic cues such as tone, pauses, changes in speech pace, emotional inflections, and verbal emphasis. These auditory cues were consistently documented in field notes during and immediately after the conversations to capture affective responses and communication nuances. These notes served as valuable aids in recalling specific details during the data analysis phase, which enriched the depth and accuracy of the findings. All focus group sessions were transcribed manually and verbatim by the first author immediately following each session. The transcripts were then fully translated into English by the same author to ensure consistency and accuracy in capturing cultural nuances. To enhance the reliability of the data, the first author cross‐verified the transcripts against the original audio recordings and field notes. The transcripts resulted in 398 pages of single‐spaced data, formatted in 12‐point font. Transcripts were then imported into Excel, where they were organized, coded, and analyzed to identify emerging themes.

## 5 Data Analysis

An inductive thematic content analysis was performed, in which emergent themes were data‐driven. Using a grounded theory approach, the analysis began with immersion in the data—listening to recordings, transcribing, and repeatedly reading transcripts to identify key categories and patterns arising directly from participant responses [42]. We initiated data analysis by carefully reading each line of the transcripts and performing open coding, an analytical process of breaking down the data into meaningful units [42]. Each line or sentence was assigned a small, descriptive unit that captured its essence. For example, the statement, “You hear or see this kind of thing yourself, like the [doctors] told my brother it is a hundred percent a cancer then open him up and say no there is nothing here! Are you kidding me?” was assigned the open code of “incorrect diagnosis.” Once conceptual labels were established through open coding, we compared the emerging codes and grouped similar concepts together, consolidating them under broader categories in the axial coding phase [42]. Categories were then organized based on patterns, ideas, and relationships observed within the data. In some cases, we refined category names to better encapsulate related units (because certain units shared connections that warranted a new category label). Finally, we identified a core category by addressing the question: *What do all these categories signify?* [42] This process was part of selective coding.

## 6 Trustworthiness and Triangulation

Following Lincoln and Guba's guidelines [43], we took detailed notes on participants' body language, emotions, and other relevant behaviors during each focus group session. Additionally, we conducted follow‐up conversations with two participants (one Afghan and one Iranian) after completing data analysis to verify that our findings accurately reflected their experiences to support triangulation. These follow‐ups strengthened the credibility of our findings by incorporating participant validation as an additional analytic check [43, 47].

The first author, an Iranian fluent in Farsi, was able to capture cultural nuances throughout the focus group sessions. For example, she noted head nodding as a sign of agreement and raised eyebrows while making the “nooch” sound as a common sign of disagreement in Iranian culture.

## 7 Results

The majority of the participants with LEP were over the age of 60 and female (Table 2). The experiences of the participants that shaped their dissatisfaction with the U.S. healthcare system were categorized into two main categories and eight subcategories. Here, we use direct quotations from participants to describe their dissatisfaction with the U.S. healthcare system and the factors that contribute to it.

**TABLE 2:** Participants' characteristics.

Nationality	6 Afghan, 19 Iranian
Mean age	65.6 years
Gender	5 males, 20 females
Regular source of healthcare provider	11 used family doctors, 4 used urgent care, 2 used relatives who are doctors, 8 had no regular source of care
Insurance	18 had insurance, 3 did not have insurance, 4 did not answer

Determinants of mistrust were grouped into system and individual factors, each with four themes.

### 7.1 System Factors

*Discrimination*: While the majority of participants initially expressed satisfaction with the U.S. healthcare system, a shift occurred when they were prompted to share personal experiences. Many recalled instances of discrimination, predominantly linked to language barriers, which significantly influenced their perceptions of the healthcare system. For instance, Sanam, a 66‐year‐old Iranian immigrant who had been in the United States for 6 years, recounted an experience in the emergency department:

My children took me to the ER [Emergency Room], but they [the doctors] didn't see me. They just said, “Wait, we have to find a translator.” My son offered to translate, saying, “I can do the translation,” but they insisted, “No, we need our own translator.” I swear, I was waiting in the ER with my children for five, maybe six hours, before they even saw me.

Similarly, Zahra, a 60‐year‐old from Iran, nodded and said, “Yes, it was the same for me. I went to the doctor, and he said, ‘Wait, I have to bring a translator,’ then he disappeared for two‐three hours. But same doctor was good with other patients waiting there.”

*Cost of Care*: A majority of participants expressed dissatisfaction with the high cost of care in the United States, noting its impact on their relationship with healthcare providers and the system overall. They viewed the expense as a significant barrier to receiving meaningful treatment, often perceiving it as driven by financial motivations rather than patient well‐being. For example, Natasha, a 67‐year‐old recent immigrant from Iran who arrived in the United States through the Diversity Visa program a year ago, shared, “Here it costs you so much just to see a doctor for a simple thing like flu. I don't even go to the doctor here…and I am healthy too, so I don't need to see the doctor, but if I have to, then I think twice.” Similarly, Majid, an Iranian who has lived in the United States for 9 years, said, “Here they ask for extra tests and stuff because they [healthcare providers] will benefit from it—I mean financially.”

*Complexity of the U.S. Healthcare System*: Most participants began their discussions by expressing frustration with the bureaucratic and complex nature of the U.S. healthcare system. They often compared these experiences to those in their home countries, where accessing care was simpler. Routine tasks, such as doctor visits and medication refills, were perceived as particularly challenging, especially given the added difficulty of navigating an English‐dominant, bureaucratic system while facing language barriers. For instance, Latifa, a 52‐year‐old Afghan immigrant who has lived in the United States for over 3 years, shared:

I had to ask my son to call the doctor many, many times to get a refill for my blood pressure medicine, and they were telling us that they told the pharmacy [to refill it], but we call the pharmacy, and they tell us, “No [there is no order from the doctor].” This kind of thing is so annoying here.

Abbi, who immigrated from Iran a year ago, agreed with Latifa, adding, “Here they love calling each other for no reason. This is a waste of time. In Iran, you just go and get your medications, but here, no, so many places should approve that medication.” Similarly, Natasha (67 years old) described her struggle to see a specialist:

I just wanted to see a pain doctor and had to go to three other [general practitioners] to finally get a referral, but in Iran, you just walk in [to the doctor's office], even though nowadays it has become such a long waiting time even in Iran.

*Pharmaco‐centric care preference*: For the purpose of this study, we define *pharmaco‐centric* as a clinical orientation that prioritizes medication‐based interventions, often at the expense of addressing patients' broader physical, emotional, social, and cultural needs. Thirty‐five percent of participants observed that the U.S. healthcare system is heavily reliant on pharmaceutical treatments, often as the primary approach to care. Many participants, particularly those who had immigrated within the last 5 years, expressed a preference for herbal remedies and home treatments for managing conditions like chronic pain, upset stomach, colds, and the flu. For instance, Hamid, a 67‐year‐old Iranian who immigrated to the U.S. 2 years ago, share the following:

I use chamomile tea and lemon or other herbal tea when I get a cold or, you know, sometimes if anyone gets diarrhea and stuff, and it helps a lot. But here my son took my grandkid to the doctor, and they gave the little kid so many medicines to take… I was shocked.

Similarly, Latifa (52 years old) expressed her preference for non‐pharmaceutical remedies: “I take Tylenol and stuff for my migraine pain, but the best is cold water; it helps me a lot. But doctors never say that, they just say take the Tylenol and Advil.”

### 7.2 Individual Factors

*Cultural belief*: Most participants subconsciously viewed their doctors as figures of authority and power, a perspective rooted in their cultural backgrounds where questioning a doctor's recommendations is uncommon. This cultural norm influenced their interactions within the U.S. healthcare system, where they tended to comply outwardly with doctors' orders, even if they privately disagreed with the suggested treatments. While they might not voice their concerns during consultations, many chose not to follow through on certain recommendations—such as refilling medications or strictly adhering to prescribed treatments—once they left the doctor's office. For example, Soory, a 75‐year‐old Iranian immigrant who moved to the U.S. from Canada, shared:

Even there [in Canada], I did not ask any questions, why bother them and take time?… At the end, I choose which medication is really good for me and use that one.

Majid from Iran smiled at Soory's comment and described his approach to medical advice in the United States: “I say yes, yes [to the doctor's recommended treatment in the U.S.], but I [come home and] take the medicine that the doctor gave [prescribe] me in Iran.”

*Cross‐contextual comparison*: Immigrants with LEP in this study frequently engaged in comparative evaluations between the U.S. healthcare system and the systems in their home countries (Iran and Afghanistan). Having spent much of their lives abroad, they brought extensive prior experience with healthcare in their native countries, which shaped their perceptions during each encounter in the United States.

For example, Leila, a 59‐year‐old who immigrated to the U.S. 1 year ago, appreciated the time and attentiveness of U.S. doctors:

It's so good that the doctor actually spends time with you and listens to you, even though they use interpreters. It makes me feel good. But in Iran, they never listen to what I say. Doctors just write the prescription and say, “Okay, go. Next patient.”

In contrast, Abbi expressed disappointment in what he perceived as an over‐reliance on diagnostic tests in the United States:

You know I think actually they [healthcare providers] don't listen to you. They just tell you go do all of these blood test then come back. When I was in Iran, doctors were so good and knowledgeable. They didn't need to do hundreds of tests, blood tests, or MRIs. They could diagnose you by your symptoms. Here, doctors don't know much; they just rely on tests.

*Experiences of misdiagnosis*: Fourteen participants in the focus groups shared experiences of misdiagnosis or underdiagnosis involving themselves or their loved ones within the U.S. healthcare system. However, four participants continued to hold a positive view, believing that the U.S. healthcare system provides the best available services. For instance, Abbi, an immigrant from Iran, recounted a distressing experience: “They told my son that he had cancer, but later said it wasn't cancer. It took three weeks to confirm it was a non‐cancerous tumor, but the stress we went through during that time was unforgivable.” Roya, from Afghanistan, nodded in agreement and shared a similar experience involving her sister‐in‐law:

My sister‐in‐law was pregnant, and they said the child is sick [had an abnormality] and you know she has to get an abortion. The poor lady was so stressed, crying every day until the next appointment when the doctor said, “Oh, sorry, that was a mistake; the baby is fine.” You know, that amount of stress is not even good for a pregnant woman.

*Linguistic miscommunication*: All participants in this study had LEP. Half of the participants in each focus group identified language as a significant barrier that affected their ability to access health information, communicate with doctors, read medication labels, and understand instructions from healthcare providers. Conversely, the other half of the participants expressed satisfaction with the availability of interpreters, which they felt improved their healthcare experience. For example, Hamid noted a positive experience with interpretation services: “They always provide me with interpreters. I think it's a California thing, I don't know. They are very good at translation.” In contrast, Fatemeh, who resides in Pennsylvania, expressed dissatisfaction:

It's not easy here in Pennsylvania. They give us an iPad, and a man on the other side translates, which is so bad and makes me feel uncomfortable. I wish we had more Farsi‐speaking doctors here. I think it differs from place to place. For example, my sister in New York, she gets good services and loves it there.

## 8 Discussion

**FIGURE 1:**
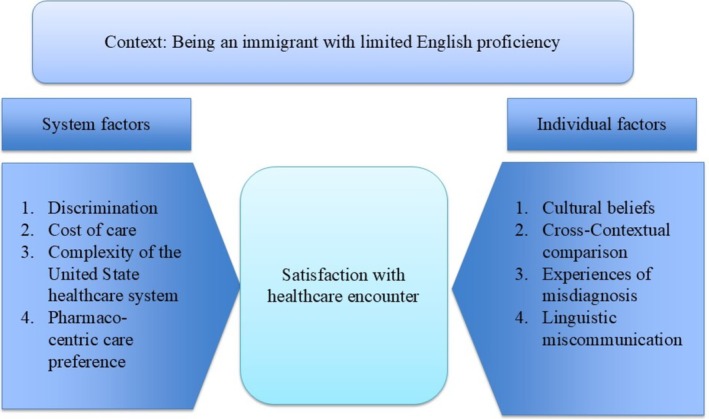
Summary of findings: Factors that negatively affect the satisfaction of LEP patients with care.

In this study, we expanded on the existing literature examining factors influencing the satisfaction among immigrant populations with U.S. healthcare [38, 48]. Specifically, we focused on the underserved and understudied population of immigrants with LEP who face unique challenges resulting from language barriers in nearly every healthcare encounter. Our findings (Figure 1) revealed that, within the LEP immigrant population, elements that negatively affect overall satisfaction with care often go unrecognized and unaddressed. These elements stem from several key factors, including cultural upbringing, experiences of discrimination, the high cost of care, the complexity of navigating the healthcare system, and a perceived over‐reliance on diagnostic tests and medication‐based treatments within the U.S. healthcare system.

Interestingly, while the majority of participants initially expressed high satisfaction with the U.S. healthcare system, deeper exploration of their personal experiences revealed significant dissatisfaction at the individual encounter level. This dissatisfaction was driven by persistent comparisons between their current healthcare experiences and those in their home countries, cultural norms they were accustomed to, instances of misdiagnosis, and language barriers.

Although language barriers were a major subcategory in this study, participants generally reported satisfaction with the interpretation services provided by the U.S. healthcare system [49, 50]. Their main concerns instead revolved around a lack of health literacy, including their inability to search for health information online in English or comprehend instructions provided by healthcare professionals during discharge, physical therapy, or medication usage. Leveraging artificial intelligence to translate medication instructions into less commonly spoken languages could offer a cost‐effective solution for pharmacists to provide accurate guidance [51]. Such innovations could improve medication adherence and enhance patient satisfaction with care.

Although there is considerable pressure to reduce medical errors in the U.S. healthcare system, some errors remain unavoidable [52]. Many immigrants with LEP have either personally experienced or heard stories of loved ones being misdiagnosed or underdiagnosed [53]. This underscores the urgent need to focus on reducing medical errors in order to increase trust in healthcare and ultimately improve patient satisfaction with care.

Afghan and Iranian participants in this study shared similar views across all questions discussed during the focus groups, with no discernible differences based on cultural backgrounds. All participants identified the high cost of care as the primary barrier to accessing healthcare and a significant factor that negatively affected their overall satisfaction with care. Many expressed difficulty understanding the rationale behind various medical procedures (e.g., blood tests, imaging). These findings align with those of Rivers and Glover, who also identified the cost of care as a key determinant of patient satisfaction [54]. Similarly, Wahlster et al. found that access to high‐cost medicines is hindered by systemic barriers, including healthcare system affordability and patient out‐of‐pocket costs. These barriers can lead to disparities in access to treatment [55].

The majority of participants expressed concerns about the U.S. healthcare system's heavy reliance on pharmacological treatments and how this negatively affects their overall satisfaction. They felt that healthcare providers often lack a holistic view of patients, treating them as one‐time transactions where a single medication is expected to resolve their issues. This sentiment aligns with the findings of Jeffs et al. who noted that fostering a holistic approach in the healthcare system enhances patient engagement and increases satisfaction with care [56]. Traditional biomedical models are criticized for being linear and reductionist. A holistic model, such as the biopsychosocial approach, considers multiple interrelated components (i.e., personal, social, and environmental factors) to provide comprehensive care [57].

This study explores elements that negatively affect overall satisfaction with the U.S. healthcare system among immigrants with LEP, focusing on Farsi‐ and Dari‐speaking populations. Our research adds a new dimension to the existing literature on healthcare satisfaction by presenting a nuanced perspective. While previous studies often characterize immigrants as being generally dissatisfied with healthcare services [38, 52], our findings reveal a different experience among Iranian and Afghan immigrants with LEP. Participants in our study consistently reported overall satisfaction with the U.S. healthcare system and a higher level of trust in its services.

This satisfaction and trust may stem from a combination of comparative tendencies, language barriers, and cultural norms. Participants expressed appreciation for the structured nature of the U.S. healthcare system, which allowed them to anticipate the process and manage expectations for each medical visit [58]. This contrasts with the often unpredictable and chaotic healthcare systems in Iran and Afghanistan [59, 60]. Their LEP may also contribute to a superficial sense of satisfaction, as participants may not fully comprehend the instructions provided, creating a perception of comfort within an English‐dominant healthcare setting despite an incomplete understanding of their encounters [61]. Participants' comparisons with healthcare services in their home countries consistently highlighted the U.S. system as more disciplined and technologically advanced [62]. Furthermore, cultural perspectives may also play a role in perceived satisfaction. Immigrants often view healthcare providers as authoritative figures who make decisions on their behalf, while their role is to comply without question [63]. This cultural expectation, combined with limited language skills, may reinforce the perception of providers as unquestionable authorities, thereby fostering a sense of satisfaction rooted in deference rather than full comprehension or genuine trust.

Notably, our participants identified several elements that negatively influenced their overall satisfaction with care, particularly during direct encounters with healthcare providers. Despite these concerns, they expressed remarkable satisfaction with the U.S. healthcare system as a whole, highlighting its perceived strengths and benefits.

## 9 Policy Implications

Achieving the Healthy People 2030 objectives of reducing health disparities requires addressing the root causes of factors that affect satisfaction with care, particularly among ethnic minorities [40]. By fostering trust and improving patient engagement, healthcare systems can better support these communities and advance health equity [64]. Central to this effort is the implementation of comprehensive cultural competency training for medical students and residents, equipping future healthcare providers with the skills to build trust and establish meaningful relationships with patients [65]. Such training is not just about bridging language gaps but also about understanding the cultural values, experiences, and expectations that shape patients' perceptions of care.

It is important to acknowledge that the demanding schedules of healthcare providers often limit the time they can dedicate to individual patients, particularly those with LEP [66, 67]. Allocating additional time for encounters with LEP patients—integrated into providers' clinical workflows—may enhance communication effectiveness, reduce misunderstandings, and ultimately contribute to greater patient satisfaction with care [18, 19].

Equitable access to healthcare must be prioritized for all individuals, regardless of immigration status or language proficiency. Policy initiatives should focus on creating healthcare environments where individuals with LEP feel valued, respected, and safe. This involves not only ensuring access to interpreters and translated materials but also fostering a culture of inclusivity within healthcare institutions. By addressing these unique needs, we can dismantle systemic barriers, foster trust in the healthcare system, and significantly reduce health disparities. Such efforts are crucial for advancing equity and ensuring that every individual receives the quality of care they deserve, ultimately improving patient satisfaction with the care.

## 10 Limitations

While this study successfully engaged hard‐to‐reach Iranian and Afghan immigrant populations with LEP, several limitations must be acknowledged. First, the focus on Farsi‐ and Dari‐speaking participants may limit generalizability to other ethnic minorities facing language barriers. Future research should include diverse linguistic and cultural groups to enhance applicability.

Additionally, while focus groups facilitated interactive discussions, they may have overshadowed individual experiences or favored consensus over depth. Some participants may have dominated discussions, potentially marginalizing quieter individuals.

Finally, conducting sessions via FaceTime or phone calls may have influenced participant comfort and response authenticity. The mixed format also limited access to body language and cultural nuances, particularly for phone participants, potentially affecting engagement and interpretation of experiences. It is important to acknowledge that the focus group design may have influenced participants' responses, as some individuals might have been swayed by others' comments or may have felt hesitant to share dissenting views in a group setting.

## Conflicts of Interest

The authors declare no conflicts of interest.

## Data Availability

Data sharing is not applicable to this article as no new data were created or analyzed in this study.
